# Comparative effects of suspension-based and dynamometer-based isometric training on neuromuscular performance in trained male athletes: a randomized comparative study

**DOI:** 10.3389/fphys.2026.1959307

**Published:** 2026-10-06

**Authors:** Mete Berk Demiryol, Mehmet Vural, Faruk Akçınar, Kadir Tiryaki, Fırat Akcan

**Affiliations:** 1 Department of Sport Management, Faculty of Sport Sciences, Gaziantep University, Gaziantep, Türkiye; 2 Department of Coaching Education, Faculty of Sport Sciences, Gaziantep University, Gaziantep, Türkiye; 3 Department of Coaching Education, Faculty of Sport Sciences, Inönü University, Malatya, Türkiye; 4 Department of Physical Education and Sport Teaching, Faculty of Sport Sciences, Düzce University, Düzce, Türkiye

**Keywords:** athletic performance, functional strength training, isometric strength, muscle balance, suspension training

## Abstract

**Objectives:**

Isometric strength training is widely used to enhance athletic performance and support rehabilitation. However, few studies compare the effects of dynamometer-based and suspension-based isometric training on performance, and the effects of these methods remain unclear. This study compares the effects of dynamometer-based and suspension-based isometric training on muscle asymmetry, balance, estimated maximal strength, anaerobic power, and isokinetic strength in trained male athletes.

**Methods:**

This study included 16 healthy male athletes aged 18 to 21 years (mean age: 18.81 ± 1.16 years; height: 178.06 ± 4.65 cm; body weight: 74.25 ± 4.32 kg; BMI: 23.43 ± 1.24 kg/m^2^; body fat: 14.29 ± 1.55%). Participants were randomly assigned to either the TRX-based isometric training group (TRG, n=8) or the dynamometer-based isometric training group (DTG, n=8). Both groups performed isometric strength training based on the principle of progressive overload for eight weeks. The TRG group completed a predetermined series of four exercises three days per week. In contrast, the DTG group performed 6-second isometric contraction series in both flexion and extension positions for each leg three days per week. We assessed data normality using the Shapiro-Wilk test and calculated the mean and standard deviation. The relationship between pre-test and post-test measurements in the TRG and DTG groups was analyzed using a two-way repeated-measures analysis of variance (ANOVA; time × group); the level of statistical significance was set at p <.05.

**Results:**

A significant main effect of time from the pretest to the posttest was found for the parameters of isokinetic peak torque, OSI, MLI, peak power, average power, and estimated 1RM (p <.05). In contrast, no significant main effect of time was observed for muscle asymmetry, API, and time to reach peak power (p>.05). Furthermore, it was stated that neither the group effect nor the time × group interaction showed a statistically significant effect on any of the performance parameters (p>.05).

**Conclusion:**

In conclusion, while an eight-week TRX and dynamometer-based isometric training program resulted in significant changes in selected strength, balance, and anaerobic power parameters attributable to the main effect of time, no significant changes were observed in group effects or time × group interactions for any of the measured parameters.

## 1 Introduction

Training models aimed at performance development in sports science are constantly evolving, alongside advances in biomotor characteristics, the application of different loading principles, and an increase in the variety of equipment (Stone et al., 2022). Strength training can induce physiological and neuromuscular adaptations through different types of muscle contractions (isotonic, isometric) against resistance encountered by the muscles (Rong et al., 2025). It has been reported that isometric strength training increases muscle strength and endurance, improves neuromuscular coordination, enhances strength gains at specific joint angles, and has positive effects on balance and postural control (Widodo et al., 2022). Furthermore, isometric loads can increase strength production capacity through neural adaptations in motor unit activation, firing frequency, and intermuscular coordination. Isometric training can also induce significant adaptations in tendon stiffness, joint-angle-specific torque production, and strength-time characteristics (Oranchuk et al., 2019; Ghayomzadeh et al., 2025).

In particular, isometric exercises performed using suspension systems such as TRX, Redcord, and Sling have been shown to contribute to the development of balance, stabilization, proprioceptive control, and functional strength (Harbili et al., 2022; Yalfani et al., 2023), while isometric exercises performed on an isokinetic dynamometer are reported to be effective in enhancing strength production, muscle balance, and angle-specific strength development (Moghadasi et al., 2024). It is believed that the instability inherent in suspension systems may increase stabilizer muscle activation, proprioceptive feedback, and intermuscular coordination. In contrast, isokinetic systems may facilitate strength adaptations based on controlled and high-torque production. Studies have reported that isometric training performed with various pieces of equipment may have more positive effects on certain performance parameters compared to traditional bodyweight isometric exercises (Gaedtke and Morat, 2016).

In particular, whether motor unit activation patterns resulting from the inherent imbalance in suspension systems lead to different adaptations than those from isokinetic exercises based on strength production at fixed joint angles remains a significant gap in the literature. Furthermore, these two basic devices differ not only mechanically but also in terms of applicability, accessibility, and ease of use. Isokinetic dynamometers ensure controlled strength production under standardized conditions. However, several factors may limit use, including accessibility, cost, size, duration of use, and technical expertise (Dvir and Müller, 2020; Wilk et al., 2024). In contrast, suspension systems offer a more practical, user-friendly option for training environments (Mozafaripour et al., 2024). Therefore, directly comparing the effects of training programs based on these two approaches on performance outcomes can provide scientifically grounded information to help coaches and athletes select a training method. Accordingly, this study aims to compare the effects of eight-week dynamometer- and suspension-based isometric training programs on balance, anaerobic performance, muscle asymmetry, estimated maximal strength, and isokinetic performance in trained male athletes. We hypothesize that TRX-based isometric training will lead to greater improvements in balance and muscle symmetry parameters because imbalance increases proprioceptive and stabilizer muscle activation. In contrast, it is believed that isokinetic isometric training will result in more pronounced adaptations in maximum strength and isokinetic performance parameters due to controlled torque production.

## 2 Materials and methods

### 2.1 Participants

Our study was designed as a randomized, two-group, pretest-posttest experimental design. The sample size was determined based on isokinetic knee extensor peak torque data obtained from a study conducted in 2005, as a result of an *a priori* power analysis performed using the G*Power 3.1.9.7 program. Using the pre-test–post-test standardized effect size (dz = 0.7684) obtained from the relevant study as a reference, the “t-tests, Difference between two dependent means (matched pairs)” model was used in G*Power; a 5% error margin (α = 0.05) and 80% power (1–β = 0.80) were assumed for the two-tailed test model. The minimum sample size was 16 (Symons et al., 2005). To assess the sensitivity of the current sample to detecting the group × time interaction, we conducted an “F-tests, (ANOVA). In the sensitivity analysis conducted using the repeated measures, within-between interaction model, it was stated that, given the current number of participants and the accepted power conditions for two groups and two measurement time points, and assuming a correlation between repeated measures ranging from 0.30 to 0.70, the minimum detectable effect size would be between f = 0.376 - 0.574. Our study included 16 male athletes with at least 6 years of active sports experience, who had participated in national-level competitions and engaged in active training at least 3 days a week (mean age: 18.81 ± 1.16 years; height: 178.06 ± 4.65 cm; body weight: 74.25 ± 4.32 kg; BMI: 23.43 ± 1.24 kg/m^2^; body fat percentage: 14.29 ± 1.55%) (**Table 1**). Before participating in the study, participants reported performing regular strength and resistance exercises as part of their routine training, not participating in any structured or systematic isometric strength training program, and continuing their routine training during the experimental training period. The inclusion criteria for the study were as follows:

**Table 1:** Demographic characteristics of participants.

Group	n	Age (years)	Height (cm)	Body mass (kg)	BMI (kg/m^2^)	Body fat (%)
		Mean ± SD				
TRG	8	18.12 ± 0.83	175.25 ± 4.71	71.50 ± 2.32	23.31 ± 1.23	13.84 ± 1.32
DTG	8	19.50 ± 1.06	180.87 ± 2.47	77.00 ± 4.17	23.54 ± 1.32	14.33 ± 1.81

- Male, aged 18–24 years;
- Engaging in regular training at least 3 days per week;
- Having at least 6 years of active athletic history.

The exclusion criteria for the study were:

- No significant lower extremity orthopedic pathology requiring clinical treatment;
- A history of lower extremity surgery within the past 3 years that resulted in a prolonged absence from regular training, or a history of serious musculoskeletal injuries to the lower extremities (such as ACL injury/reconstruction, severe ligament or meniscus injuries, or fractures) (Bahr et al., 2020).

### 2.2 Experimental design

Before the experiment, all participants were informed about the laboratory visits and the study protocol. To collect the data obtained in the study, the tests were administered in two sessions: a pretest and a posttest under appropriate controlled laboratory conditions. Participants visited the laboratory a total of five times during the study. During their first visit, after receiving the necessary information, they took anthropometric measurements and were introduced to the measuring devices, testing machines, and training equipment. Participants were also instructed to eat their last meal at least 3 hours before each test, to avoid any ergogenic aids (caffeine, etc.) or supplements that could affect the study (creatine, BCAAs, etc.) for 12 hours, and to refrain from intense physical activity for 48 hours before the tests. Before the pretest measurements, participants completed a one-week familiarization period to become accustomed to the isometric training protocols and equipment. During this period, the participants completed two sessions. They used the TRX suspension system during the second visit and the isokinetic dynamometer during the third visit. The researcher first explained the exercises and proper technique and then demonstrated them hands-on. The researcher took necessary precautions to ensure all participants followed the same procedure, and each participant performed the relevant isometric exercise under controlled conditions.

During their fourth visit, at least 48 hours after completing the familiarization period, participants’ balance and anaerobic power performance measures were assessed; during their fifth visit, maximum strength (estimated 1 RM) and isokinetic strength performance measures were assessed. After collecting pre-training data, participants were randomly assigned to one of two groups using a simple computer-based randomization method: the TRX-based isometric training group (TRG, n=8) and the dynamometer-based isometric training group (DTG, n=8). We did not stratify based on the demographic characteristics collected during randomization. We randomized participants using a random number generator formula in Excel, ensuring each participant was assigned to either group with equal and independent probability. To minimize differences among participants, we established eligibility criteria of age (18–21 years), sports experience (at least 6 years), and regular training participation (at least 3 days per week). To prevent assignment bias, a researcher not involved in the evaluation assigned groups. Furthermore, to prevent potential bias during testing, the researchers did not disclose participants’ group assignments to the researchers conducting the measurements (Lim and In, 2019). Additionally, all participants, who were randomly assigned to groups, remained in the study until the final analysis, with no loss to follow-up or withdrawal during the training period (**Figure 1**). Participants assigned to the TRX-based isometric training group (TRG) performed isometric strength training with TRX equipment for 8 weeks; this training consisted of 4 exercise sets, 3 days a week, involving isometric contractions in a standing or supine position in accordance with pendulum and balance principles. Participants assigned to the dynamometer-based isometric training group (DTG) performed 6-second isometric contractions on an isokinetic dynamometer three days a week for 8 weeks, with specific repetition counts and ranges of motion. At their sixth visit, 48 hours after the final day of the 8-week training period, participants’ balance and anaerobic power performance outcomes were measured during the post-test assessments; the study concluded at their seventh and final visit with the measurement of maximum strength (estimated 1 RM) and isokinetic strength performance outcomes.

**Figure 1:**
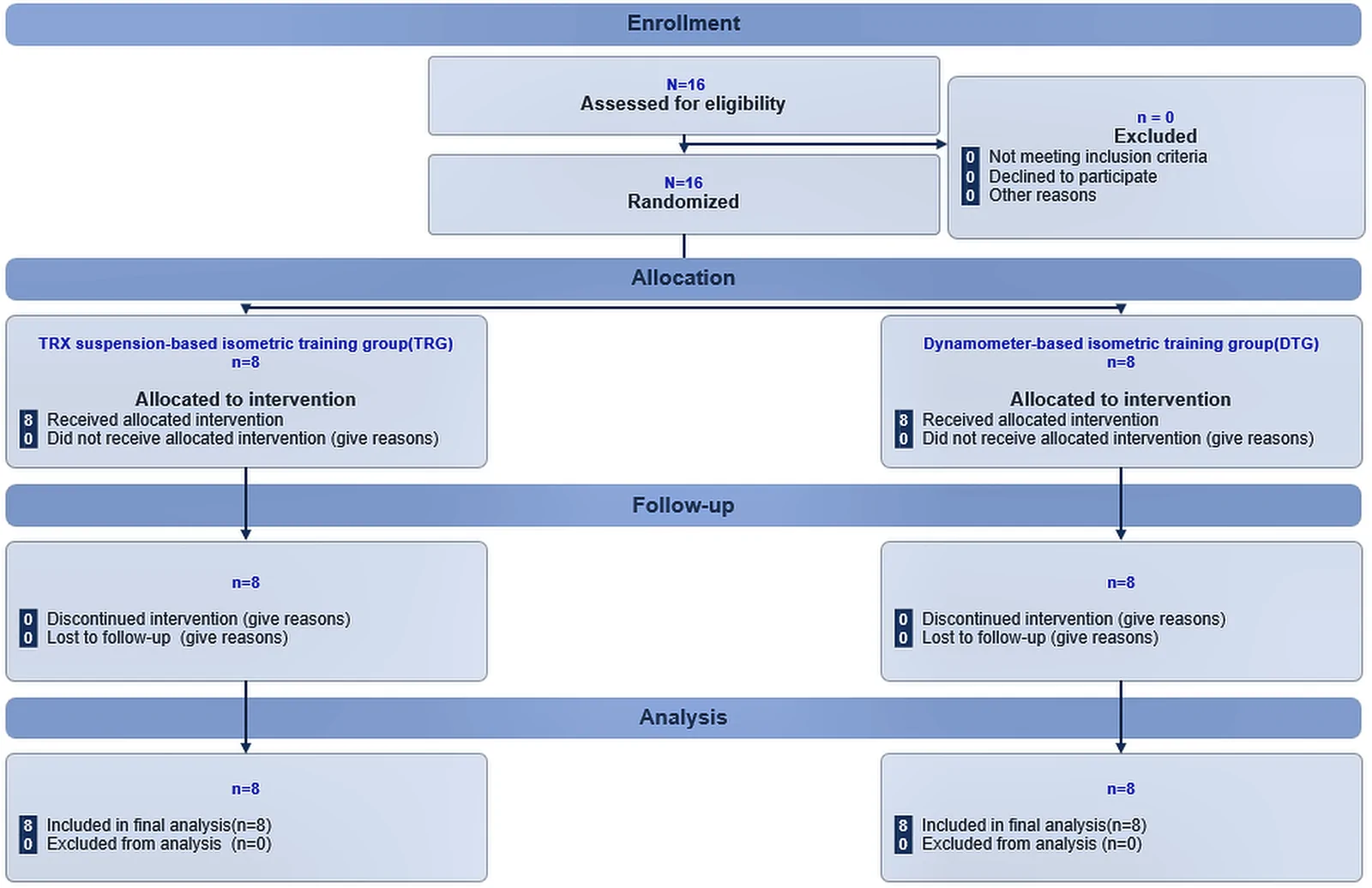
CONSORT flow.

Additionally, participants rested at least 48 hours between measurement days.

To standardize testing, measurements were administered in the same order for all participants in both the pre-test and post-test sessions. To minimize potential fatigue effects, we provided rest periods of at least 40 minutes between measurements conducted on the same day. On the first measurement day, the balance test was performed first, followed by the Wingate anaerobic power test after at least 40 minutes of passive rest; on the second measurement day, the isokinetic strength test was performed first, followed by the maximum strength test (estimated 1 RM test) after at least 40 minutes of passive rest (**Figure 2**). The same researcher conducted the pre-test and post-test performance measurements and was responsible for the laboratory and experienced in performance testing and measurement. A different researcher monitored the training process, randomization, and group assignments and was not involved in the performance measurements; this researcher also monitored the training sessions to ensure they followed the established protocol. All participants completed all 24 sessions of the eight-week training program, and the researcher monitored them for any potential injuries, pain, or adverse events.They took necessary safety precautions throughout the training process to minimize loss of progress. We obtained written informed consent from all volunteers participating in the study, and the research was approved by the Gaziantep University Ethics Committee for Sports and Health Sciences on March 17, 2025 (Decision No.: 625745/01).

**Figure 2:**
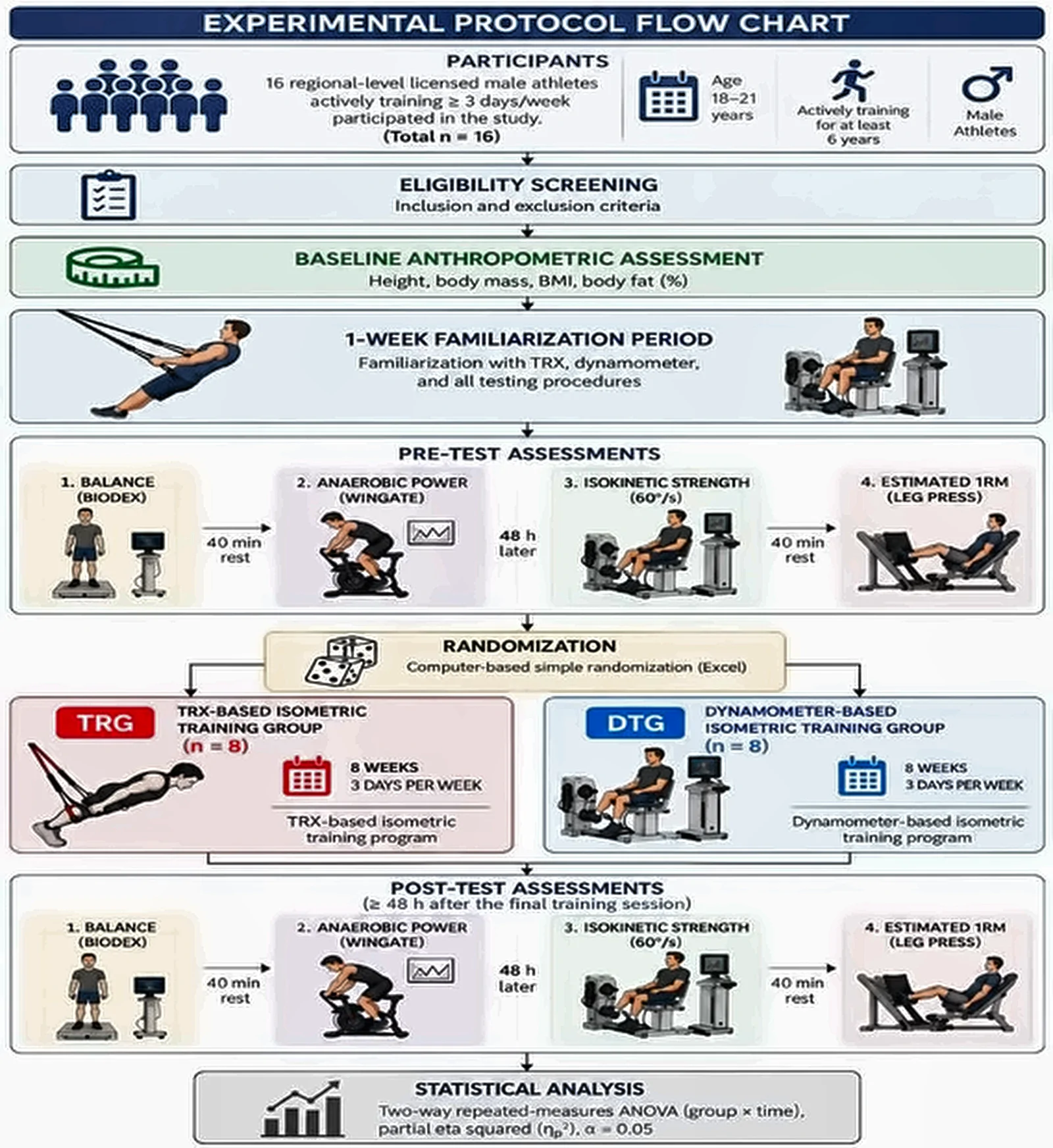
Research Protocol.

#### 2.2.1 Warm-up protocol

Before each test and training session, the athletes followed a specific warm-up protocol designed to increase muscle temperature, optimize neuromuscular activation, and reduce the risk of injury. Before the study, the participants pedaled on a stationary bike at 60 rpm for 5 minutes without resistance. They then performed 3 sets of 30 seconds of plantar stretching followed by 30 seconds of dorsal stretching before beginning the experiment or training session.

#### 2.2.2 TRX training protocol (TRX-based)

Isometric exercises were performed using TRX equipment via the suspension training method, standardized based on the pendulum and stability principles, and securely anchored to the ceiling with an appropriate suspension apparatus (Wang et al., 2025). In accordance with the pendulum principle, they maintained vertical alignment between the subjects’ center of gravity and the suspension point under control; in accordance with the stability principle, they manipulated the angle of the subjects’ bodies relative to the floor to alter balance requirements and muscle activation levels during the exercise. Participants performed four predetermined exercise sequences: TRX Squat Hold, TRX Single-Leg Lunge Hold (right–left), TRX Leg Curl Hold, and TRX Bulgarian Split Squat Single-Leg Hold (right–left) according to the principle of progressive overload [for each exercise, weekly time-based progression in isometric hold durations (a 5-second increase each week) and angular progression (body angle set at 45 degrees for the first 4 weeks and reduced to 30 degrees for the last 4 weeks)].

The intensity of the load was measured using the participants’ own body weight; to standardize body angles, we used the RateFast Goniometer app (Alchemcy Logic Systems, INC) during each training session to ensure that participants maintained the specified angle for each exercise. Initially, a 45° trunk angle was used as the baseline for all participants via the goniometer app. Participants were instructed to maintain their spines in a neutral position throughout the training and to avoid movements that would disrupt the contraction or affect its integrity during contraction (Laurenzi and Kevin, 2010). They completed the study over 8 weeks in accordance with the training program (**Figure 3**).

**Figure 3:**
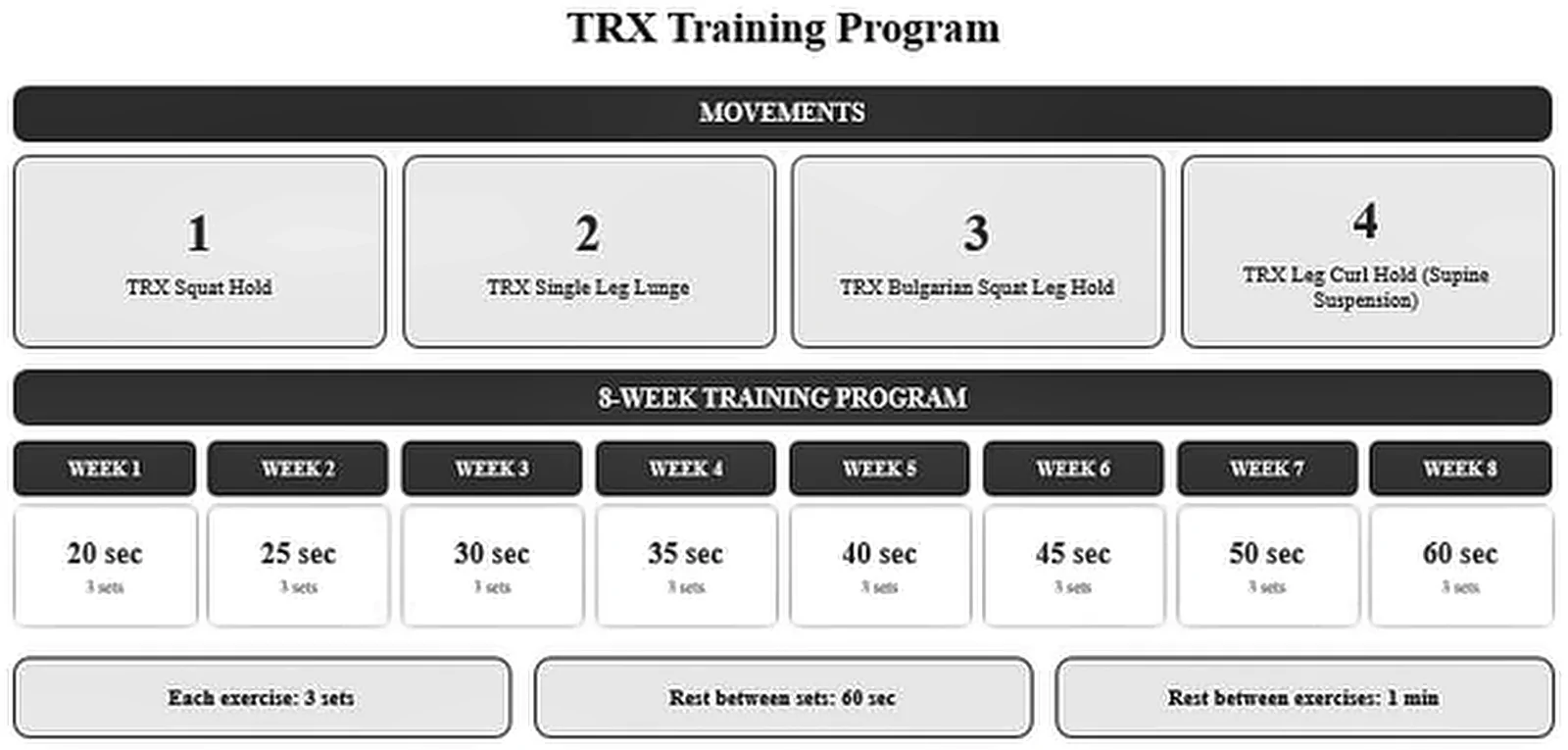
TRG exercise movements and training program.

#### 2.2.3 Isometric training protocol (dynamometer-based)

Isometric exercises were performed with the subjects seated using a CSMI-brand isokinetic dynamometer (Humac Norm Testing and Rehabilitation System, CSMI, USA) for testing and rehabilitation. In the study, DTG participants completed three training sessions per week, and during each session, they performed isometric training on both the dominant and non-dominant legs. Researchers performed Isometric contractions in the directions of knee flexion and extension for both lower-extremity legs within the same training session. During each training session, participants first performed isometric exercises on the dominant leg and then on the non-dominant leg. During training, the subjects were stabilized on the dynamometer with the knee joint at 90° of flexion and 0° of extension; we secured the pelvic and femoral segments with appropriate straps to enhance the reliability of the training. Additionally, to ensure that they performed the isometric contraction at the specified joint angle at an optimal level, the ankle apparatus was adjusted so that the tibial shaft aligned with the device’s rotational axis and the knee joint aligned with the level of the lateral condyle, thereby guaranteeing the accuracy of the contraction biomechanics (Hirano et al., 2020; Zöger et al., 2023).

Throughout all training sessions, the researcher provided verbal encouragement to help the subjects achieve maximum performance. To ensure optimal development of the subjects’ isometric contractions, the average values of the peak torque angles (joint at angle peak torque) they produced during the pretest at an angular velocity of 60°/s were used as a reference to determine the angles for flexion (right leg: 35°, left leg: 31°) and extension (right leg: 45°, left leg: 40°) were determined, and they performed isometric contractions (Stotz et al., 2022). To ensure voluntary isometric contractions using the isokinetic dynamometer, minimum threshold values were set so that participants would generate at least 150 nw of torque in the extension position and at least 100 nw of torque in the flexion position. The subjects performed the specified isometric contraction exercise for both legs according to the principle of progressive overload [weekly repetition-based progression in isometric contractions for each exercise (an increase of 1 repetition per week) and an increase in the minimum torque production threshold (150 nm of torque in extension and 100 nm of torque in flexion for the first 4 weeks; 200 nm of torque in extension and 150 nm of torque in flexion for the last 4 weeks)]. They completed the study over 8 weeks in accordance with the training program (**Figure 4**).

**Figure 4:**
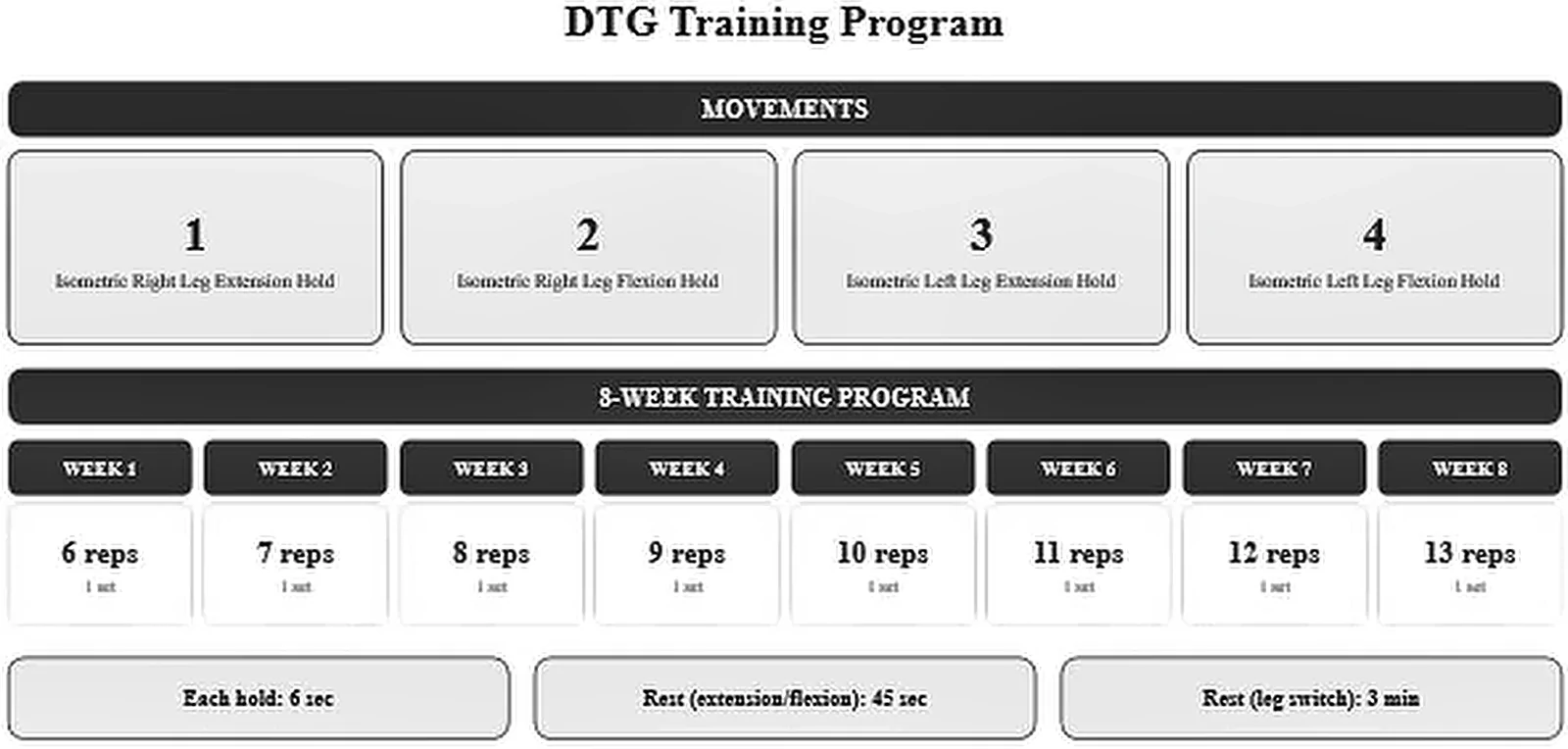
DTG Exercise Movements and Training Program.

### 2.3 Data collection tools

#### 2.3.1 Biodex balance test

Dynamic balance performance was assessed using the Biodex Balance SD system (Biodex Medical Systems Inc., Shirley, NY, USA) via a single-leg balance test on the dominant limb. During the test, participants placed their dominant foot barefoot on the device platform in a standardized foot position, kept their hands steady on their pelvis, and completed the test with their eyes open. During the test, the Biodex Balance SD system platform stability level was set to 4. Participants maintained their balance in this manner throughout three trials, each lasting 20 seconds, with 10-second rest intervals between sets, after which the test concluded. At the end of the test, following the three 20-second trials, the participants’ average overall stability balance (OSI), anterior-posterior (API), and medial-lateral (MLI) balance scores from the three trials were calculated, and the data were recorded (Blasco et al., 2023; Cachupe et al., 2001; Güler et al., 2020).

#### 2.3.2 Muscle asymmetry test

Regarding muscle asymmetry, we examined the bilateral ratios of muscles in both lower extremities. The dominant lower extremity was determined as the leg the participant preferred to use for kicking the ball. Bilateral strength asymmetry for the hamstring and quadriceps muscle groups was calculated separately. The peak torque values of the quadriceps and hamstring muscles for the dominant and non-dominant extremities, obtained from an isokinetic knee extension-flexion test performed at an angular velocity of 60°/s^−1^, were used to calculate these values. The asymmetry ratio was calculated using the formula: [(dominant limb peak torque – non-dominant limb peak torque)/dominant limb peak torque] × 100. When calculating the quadriceps bilateral ratio [Q_asym_(%)] and the hamstring bilateral ratio [H_asym_(%)], values close to 0 indicated higher bilateral symmetry, while values farther from 0 indicated differences in strength between the limbs (Tatlıcıoğlu et al., 2019).

Formula: $Asymmetry\;(\%)=\frac{\left|PT\_D\;-\;PT\_ND\right|}{PT\_D}\times 100$ PT_D: Peak torque of the dominant limb.
PT_ND: Peak torque of the non-dominant limb.

#### 2.3.3 Anaerobic power test

To measure anaerobic power, the Wingate anaerobic power test was conducted using an electronically braked bicycle ergometer (Monark Ergomedic 894E, Monark Exercise AB, Vansbro, Sweden). After the participants’ data were recorded in the system, a weight equal to 7.5% of their body weight was placed on the saddle, and the test began. To standardize the external load among participants, we determined workload settings based on body mass (W·kg^−1^). Before the test, participants completed a standard warm-up protocol: 5 minutes of cycling at 50 W, followed by two short accelerations (~5 s) to prepare for high-intensity exercise.

Following the warm-up, participants were given a 2-minute passive rest period before beginning the performance test. In the main phase, to ensure rapid attainment of maximum power output, a predetermined resistance (7.5% of body weight) to the saddle was applied at the start of the 30-second test, and verbal encouragement was provided throughout the 30 seconds to enable the subject to perform at their maximum capacity. At the end of the 30 seconds, the athlete completed the test and moved on to the cool-down phase, during which the subject continued pedaling without resistance for another 2 minutes before concluding the session (Bar-Or, 1987). The Wingate anaerobic power test consisted of a single 30-second maximal effort, and the study reported average power, anaerobic peak power, and the time to peak power output.

#### 2.3.4 Estimated 1RM maximum strength test

The participants’ maximum lower-limb strength was measured using speed-based training sensors (ENODE Pro, Enode Ltd., Finland) on a leg press machine based on an estimated one-rep maximum. Before the test, participants warmed up with dynamic flexibility exercises after 10 minutes of low-intensity aerobic exercise. After the dynamic warm-up, the starting load was set at 60% of the participant’s body weight (Liang et al., 2007). The speed-based training sensor was mounted at the midpoint of the machine’s moving part to ensure linear tracking of the machine’s movement; a tablet was used to monitor the sensors electronically, adjust the current weight by following the app’s instructions, and track measurements.

Participants performed three repetitions at the highest possible speed for each specified load. After each set of three repetitions, the weight was gradually increased, taking into account the estimated strength value tracked in the ENODE app. To ensure adequate recovery between successive strength trials for determining the estimated 1RM, participants rested passively for 5 minutes. If a participant could not perform three repetitions at the highest possible speed with the load specified in the app, the estimated 1RM value calculated from movement speed was recorded. Consequently, the study reported the estimated 1RM value rather than a directly measured 1RM. It has been reported that the ENODE Pro device used in this study provides a high degree of concurrent validity for average movement speed during resistance exercises, and that methods based on the load-velocity relationship on the leg press machine yield valid and reliable results in estimating 1RM (Achermann et al., 2023; Liao et al., 2021; Marques et al., 2021; Marcos-Pardo et al., 2019).

#### 2.3.5 Isokinetic strength test

A CSMI-brand isokinetic dynamometer (Humac Norm Testing and Rehabilitation System, CSMI, USA) was used to assess isokinetic strength performance. The subjects’ knee flexor and extensor muscle groups were assessed in both legs in CON-CON mode at an angular velocity of 60°/s^−1^ for 5 repetitions. Before the test, subjects underwent a submaximal warm-up consisting of 15 repetitions at an angular velocity of 300°/s^−1^ to familiarize them with the device (Dvir, 2004).

After a warm-up consisting of 15 repetitions, a 40-second passive rest period was observed, followed by five maximal repetitions at an angular velocity of 60°/s^−1^. During the test, the subjects were secured with stabilization straps in the trunk, pelvic, and femoral regions, and the dynamometer’s axis of rotation was aligned with the lateral femoral condyle. The highest strength value obtained was recorded as peak torque in Newton-meters. In addition, the instantaneous joint angle at which they generated this peak torque was determined as the “joint angle at peak torque, “ and the H/Q ratio was calculated to determine muscle strength balance. Throughout all test sessions, the researcher provided verbal encouragement to ensure that the subjects achieved maximum performance.

### 2.4 Statistical analysis

Statistical analyses were performed using SPSS 20.0 (IBM Corp., Armonk, NY, USA). Descriptive statistics are presented as mean ± standard deviation (Mean ± SD). The normality of the data was assessed using the Shapiro–Wilk test, and the homogeneity of variances was evaluated using Levene’s test. To evaluate the potential effects of the groups on initial body weight, isokinetic and anaerobic power data were normalized to body weight and analyzed. We applied a two-way repeated-measures ANOVA model to the resulting relative variables. To examine differences between pre-test and post-test measurements in the TRX-based and dynamometer-based training groups, we used a two-way repeated-measures analysis of variance (ANOVA; time × group). This analysis evaluated the time effect (pre-test–post-test change), the group effect (TRX–dynamometer difference), and the time × group interaction separately. We used the Bonferroni test, a multiple group comparison test, to determine statistical significance between groups and identify which group benefited from a significant difference. Partial eta-squared (ηp^2^) values were reported as measures of effect size. Effect sizes were classified as small (.01), medium (.06), and large (.14) (Richardson, 2011). Statistical significance was set at p <.05.

## 3 Results

Our data analysis revealed the following findings. First, the demographic characteristics of the participants in the TRX-based isometric training group (TRG; n=8) and the dynamometer-based isometric training group (DTG; n=8) were presented; subsequently, the collected data were examined comparatively.

**Table 2** compares pre- and post-test peak torque and bilateral muscle asymmetry in the knee flexor and extensor muscles in the TRX-based and dynamometer-based training groups, measured at an angular velocity of 60°/s^-1^. The analysis showed a statistically significant main effect of time on peak torque values for the knee flexors and extensors across all muscle groups (p <.05). In contrast, while the group effect and the time × group interaction were not found to be statistically significant, a strong effect size was observed for the time × group interaction regarding peak torque (extension) in the right leg (ηp^2^ = .140); however, when interactions in other parameters were examined, the effect sizes were found to be small (p>.05: ηp^2^ = .021 (Right_PTFLX_);.025 (Left _PTEX_);.006 (Left _PTFLX_)). Additionally, no significant effects were found for time, group, or the time × group interaction for muscle asymmetry measured by the Q_asym_ and H_asym_ ratios; however, the time × group interaction for the H_asym_ ratio was moderate (p>.05; ηp^2^ = .111). When examining mean values, we observed changes in both training groups (**Figure 5**).

**Table 2:** Comparison of pre-test–post-test isokinetic peak torque values of the knee flexor and extensor muscles at an angular velocity of 60°/s^-1^ between groups.

	PT	Group	Pre-test (Mean ± SD)	Post-test (Mean ± SD)	Time F (p)	Group F (p)	Time*Group F (p)	$\eta_{\text{p}}^{2}$ (time × group)
Right Leg	PT_EX_ (Nm)	TRG	262.63 ± 52.49	285.75 ± 53.21	23.06 (**<.001**)	.65 (.43)	2.29 (.15)	.140
		DTG	233.50 ± 47.57	277.88 ± 37.29				
	PT_FLX_ (Nm)	TRG	163.63 ± 47.40	189.50 ± 27.04	26.77 (**<.001)**	1.07 (.31)	.30 (.59)	.021
		DTG	146.25 ± 19.51	178.25 ± 13.65				
Left Leg	PT_EX_ (Nm)	TRG	238.75 ± 71.48	277.63 ± 54.60	24.62 (**<.001**)	.02 (.89)	.36 (.55)	.025
		DTG	229.50 ± 57.14	279.13 ± 53.53				
	PT_FLX_ (Nm)	TRG	135.50 ± 44.17	169.13 ± 32.86	33.11 (**<.001**)	.01 (.92)	.08 (.77)	.006
		DTG	135.50 ± 18.59	165.88 ± 33.42				
Muscle Asmmetry	Q_asym_ (%)	TRG	14.58 ± 2.74	10.31 ± .76	1.48 (.24)	1.46 (.24)	.62 (.44)	.043
		DTG	10.31 ± 1.07	10.07 ± 1.03				
	H_asym_ (%)	TRG	12.24 ± 1.10	11.32 ± .92	.712 (.41)	1.68 (.21)	1.74 (.21)	.111
		DTG	10.88 ± 1.61	10.09 ± 2.06				

PT, Peak Torque; EX, Extension; FLX, Flexion; Nm, Newtonmeter; %, Percentage; Q_asym_, Quadriceps Asymmetry; H_asym_, Hamstring Asymmetry.

p-values in bold indicate that p < .05.

**Figure 5:**
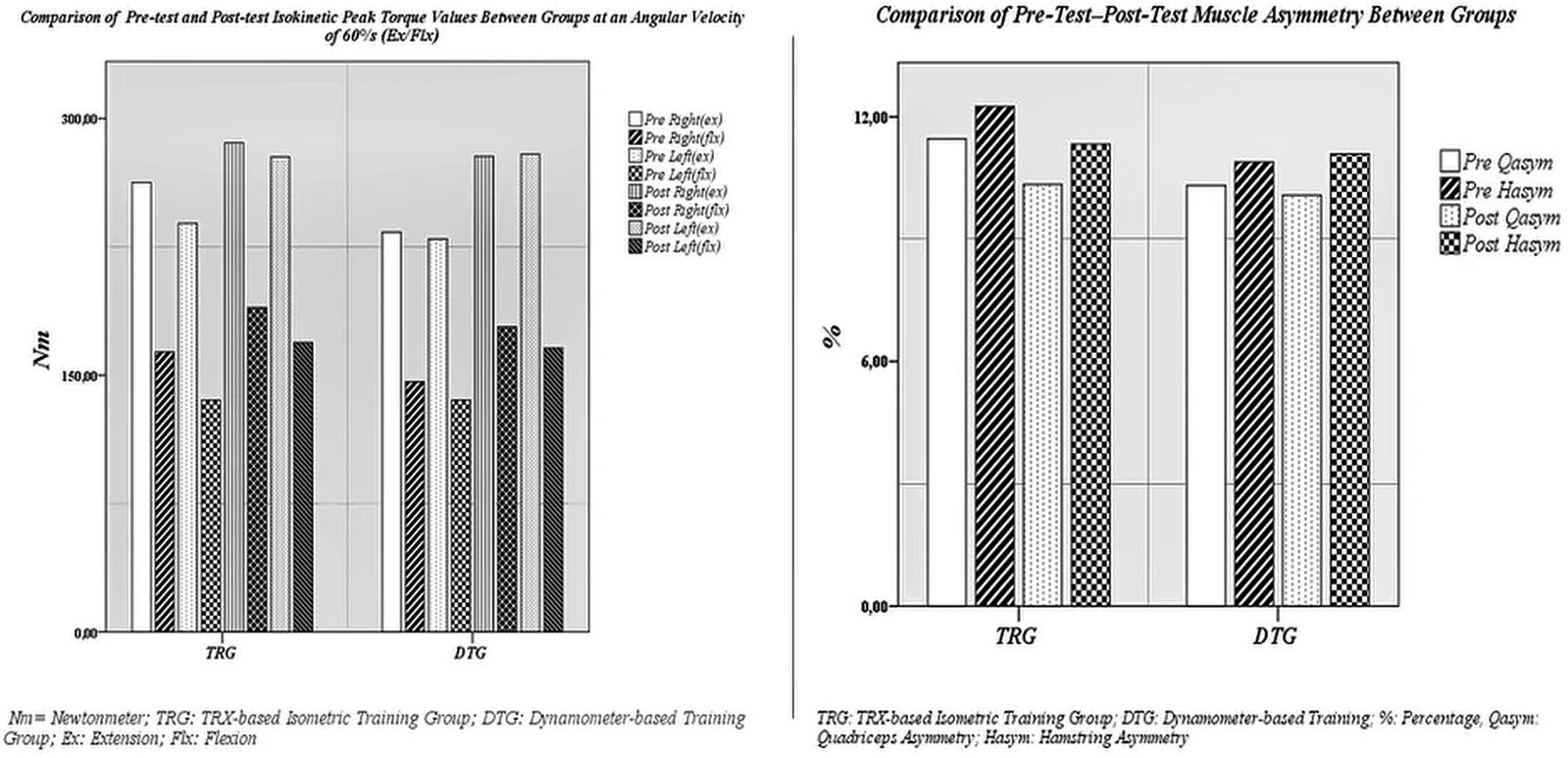
Comparison of Isokinetic Peak Torque and Muscle Asmmetry Values in Between Groups at an Angular Velocity.

**Table 3** compares pre- and post-test balance parameters (OSI, MLI, and API) in the TRX-based and dynamometer-based training groups. The analysis showed a statistically significant main effect of time on OSI and MLI parameters (p <.05); however, the group effect and the time × group interaction for both parameters were not statistically significant (p>.05). Separately, the effect size for the interaction was small for the OSI parameter (ηp^2^ = .01). For the MLI parameter, it was calculated as strong (ηp^2^ = .184). Regarding the API parameter, no significant differences were detected for time, group, or the group × time interaction, and the effect size for the interaction was small (p>.05; ηp^2^ = .04). When the mean values were examined, changes were observed in both training groups (**Figure 6**).

**Table 3:** Comparisons of pre-test and post-test balance parameters (OSI, MLI, and API) between groups.

	Group	Pre-test (Mean ± SD)	Post-test (Mean ± SD)	Time F (p)	Group F (p)	Time*Group F (p)	$\eta_{\text{p}}^{2}$ (time × group)
OSI (Score)	TRG	1.37 ± 0.35	1.05 ± 0.30	20.619 (**<.001**)	.508 (.488)	.008 (.930)	.001
	DTG	1.24 ± 0.53	0.92 ± 0.37				
MLI (Score)	TRG	1.04 ± 0.21	0.69 ± 0.27	16.662 (**<.005**)	3.536 (.081)	3.166 (.097).	.184
	DTG	0.76 ± 0.16	0.63 ± 0.20				
API (Score)	TRG	0.81 ± 0.26	0.74 ± 0.24	1.402 (.256)	.149 (.705)	.056 (.816)	.004
	DTG	0.76 ± 0.63	0.65 ± 0.29				

OSI, Overall Stability Index; MLI, Medial/Lateral Stability Index; API, Anterior-Posterior Stability Index.

p-values in bold indicate that p < .05.

**Figure 6:**
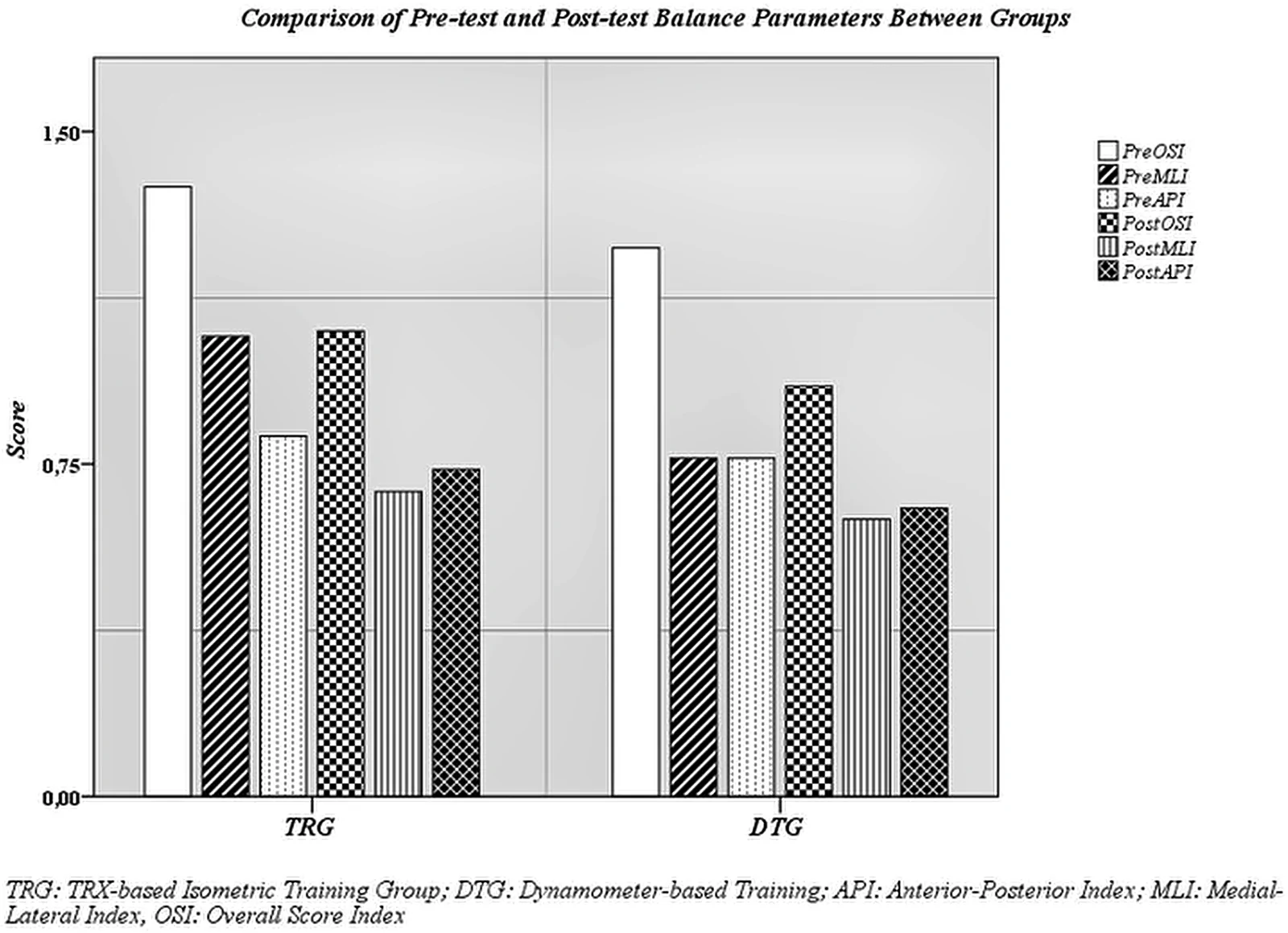
Comparison of Pre-test and Post-test Balance Parameters Between Groups.

**Table 4** compares pre-test and post-test measurements of anaerobic power and estimated 1 RM maximum strength parameters for TRX-based and dynamometer-based training programs. The analysis showed a statistically significant main effect of time on peak power and average power parameters (p <.05); however, the group effect and the time × group interaction were not statistically significant; the effect size for the interaction was small for peak power and moderate for average power (p>.05; ηp^2^ = .037_(PP)_,.073_(AVGP)_). However, no significant differences were found in the time to peak power parameter in terms of time, group, or the time × group interaction (p>.05). Additionally, a significant main effect of time on maximum strength (estimated at 1 RM) was found (p <.05); however, while the group effect and the time × group interaction were not statistically significant, the effect size was reported to be large (p>.05; ηp^2^ = .160). When we examined the mean values, we observed changes in both training groups (**Figure 7**).

**Table 4:** Comparison of pre-test–post-test anaerobic power parameter and estimated 1 RM maximal strength values between groups.

	Groups	Pre-test (Mean ± SD)	Post-test (Mean ± SD)	Time F (p)	Group F (p)	Time*Group F (p)	$\eta_{\text{p}}^{2}$ (time × group)
PeakPower (W)	TRG	838.93 ± 123.90	918.31 ± 145.31	9.190 (**<.009**)	1.390 (.258)	.536 (.476)	.037
	DTG	747.84 ± 165.85	796.34 ± 271.51				
Time to Peak Power (s)	TRG	2.15 ± 0.56	2.04 ± 0.67	.013 (.911)	.075 (.788)	.666 (.428)	.045
	DTG	1.96 ± 0.86	2.04 ± 0.86				
Avg. Power (W)	TRG	528.13 ± 134.05	601.37 ± 104.98	7.083 (**<.019**)	1.259 (.281)	1.102 (.312)	.073
	DTG	488.70 ± 81.66	520.50 ± 128.73				
Estimated 1RM Maximal Strength (kg)	TRG	210.00 ± 42.76	270.6 ± 56.97	44.853 (**<.001**)	1.031 (.327)	2.661 (.125)	.160
	DTG	197.50 ± 34.23	234.38 ± 61.84				

Avg. Power, Average Power; W, Watt; s, Second; kg, kilogram.

p-values in bold indicate that p < .05.

**Figure 7:**
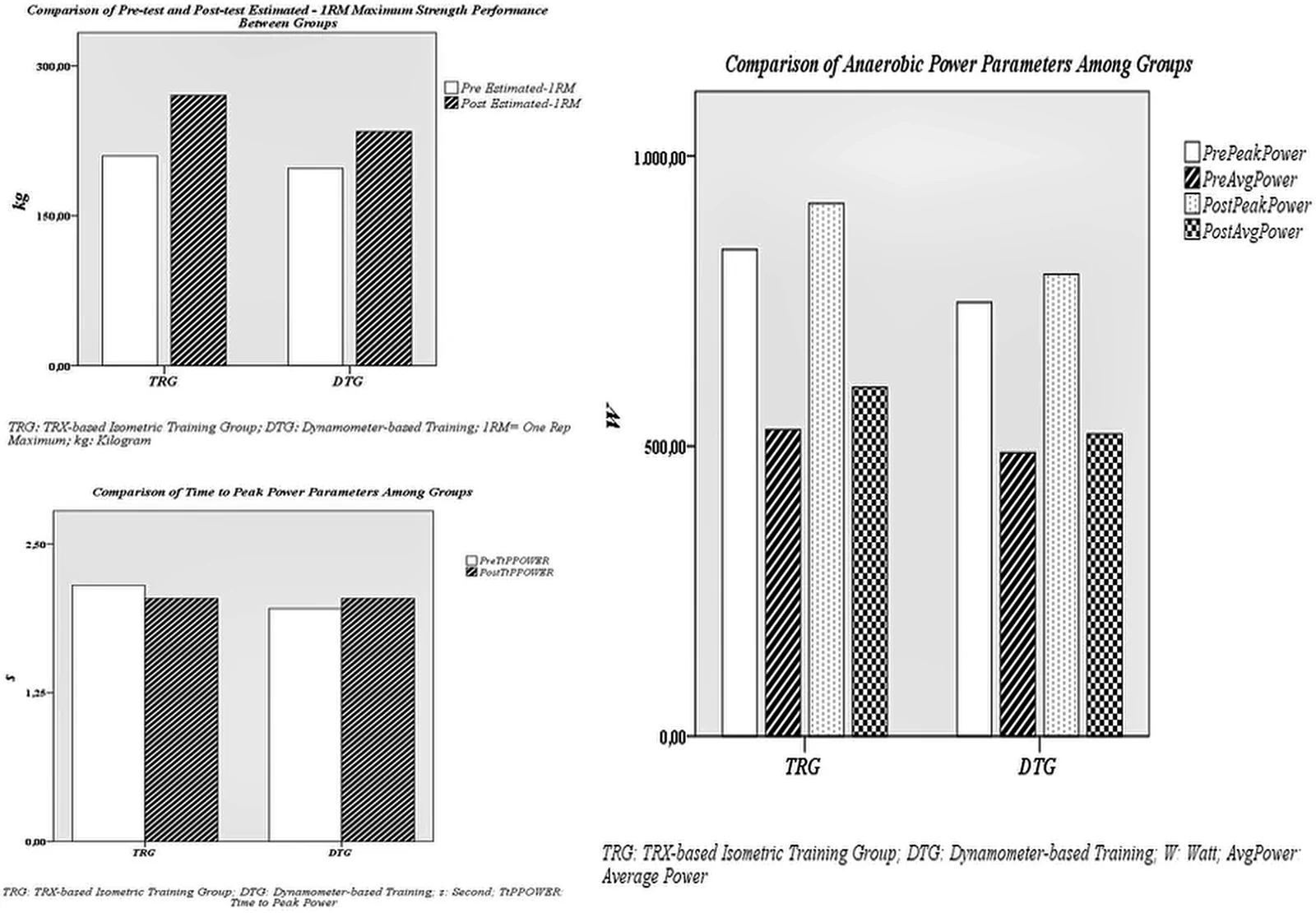
Comparison of Pre-test and Post-test Anaerobic Power Parameters and Estimated-1RM Maximal Strength Between Groups

In additional analyses evaluating the potential effect of body mass, isokinetic and anaerobic power parameters were normalized for body mass; the results in absolute values remained consistent. While a significant main effect of time was observed in the isokinetic extensor-flexor relative peak torque, anaerobic relative peak power, and relative average power data (p <.05), no statistically significant difference was found in the group effect or the time × group interaction (p>.05).

## 4 Discussion

After the 8-week isometric strength training program, time had a statistically significant main effect on OSI, MLI, isokinetic peak torque, average power, peak power, and estimated 1 RM parameters. In contrast, group and time × group interactions were not statistically significant for any variable examined. Similarly, time, group, and time × group interactions were not statistically significant for the muscle asymmetry parameter. The time-dependent changes observed in performance parameters in both groups may be related to adaptation to the repeated isometric contraction stimuli applied in both training programs. In our study, the significant main effect of time on peak torque values at 60°/s^-1^ in both the TRG and DTG groups likely stems from a stimulus that both training approaches translate into dynamic isokinetic strength production, rather than solely static strength production. A review of the literature indicates that nine weeks of isometric exercise training for the lower extremities increases isokinetic strength and yields a value close to the isometric strength level developed through dynamic training (Folland et al., 2005). A recent study reported that isometric resistance training increased isometric strength compared to a control group, while isokinetic resistance training had a more limited effect on isometric strength (Ghayomzadeh et al., 2025). It is also stated that isometric exercises have positive effects not only on static strength but also on dynamic performance outcomes, and that this effect is primarily driven by increased motor unit activation, firing frequency, and an increase in neuromuscular drive originating from the central nervous system (James et al., 2023; Vural et al., 2023). The fact that the time × group interaction was not statistically significant for isokinetic peak torque suggests that the different structural characteristics of multi-joint isometric loading in TRX -based training and joint-angle-specific isometric loading in dynamometer-based training do not systematically affect high-level strength production. That adaptation did not show a clear distinction between the groups.

When bilateral muscle asymmetry was assessed, the lack of statistical significance for time, group, and time × group interactions in both quadriceps and hamstring asymmetry values indicates that absolute strength gains did not alter the relative strength difference between the two limbs to a measurable degree. The literature indicates that both unilateral and bilateral strength training significantly increase isometric peak torque; however, neither training model significantly alters the inter-limb symmetry index (Lynch et al., 2023). Similarly, asymmetry levels should not be examined by test, training method, type of strength, or scope, as bilateral symmetry is individual-specific and does not change solely due to strength (Bishop et al., 2023). We concluded that our findings are consistent with the literature. This is because, in our study, we did not implement individualized training protocols such as specific exercises of varying intensity or those aimed at reducing asymmetry for the initially weaker limb. Consequently, strength levels in both the dominant and non-dominant limbs may have increased together, and the proportional difference may have been largely preserved.

In our study, the significant main effect of time observed in the OSI and MLI balance parameters is consistent with these findings. However, the lack of a significant change in the anterior-posterior stability index (API) may stem from the fact that dynamic balance components require higher sensitivity or longer training duration, or because balance adaptation did not generalize to all aspects of postural control. A systematic review of studies conducted with athletes reported that instability-based resistance training, including suspension training, can improve balance performance (Gao et al., 2025). However, the lack of improvement in API performance suggests that postural stability adaptations may vary by direction. The current meta-analysis reported that balance-related performance may be significantly direction- and task-specific (Kümmel et al., 2016). The fact that the time × group interaction was not statistically significant for the OSI and MLI parameters suggests that the additional instability created by TRX-based isometric training even if it imposes a higher demand for postural control during training may not directly affect the postural stability test assessed using the Biodex. The absence of balance-specific instability in dynamometer-based training should not be interpreted as impaired balance performance in this group. Indeed, resistance training can improve postural balance.

Additionally, the lack of a statistically significant time × group interaction effect for the MLI parameter, despite a large effect size, may be due to the small sample size. To detect a potential difference, this should be examined separately in larger samples. When evaluated in terms of anaerobic performance, isometric strength development is associated with the capacity for high-intensity energy production. In particular, more efficient use of the phosphagen energy system, increased motor unit activation capacity, and improved neuromuscular efficiency may explain improvements in anaerobic power output (Cormie et al., 2011; Sahlin, 2014). In our study, the significant main effect of time on peak power and average power values suggests that progressive isometric strength training may contribute to high-intensity power production. The literature well establishes that dynamic power outputs relate to isometric strength performance and that lower-limb isometric strength, in particular, is associated with cycling and anaerobic peak power (Stone et al., 2004; Kordi et al., 2020). Similarly, a resistance training study using TRX and BOSU reported significant increases in lower-limb peak and average power output (Maté-Muñoz et al., 2014). Separately, the absence of a significant change in the time to peak power indicates that, despite the magnitude of power produced after training, the rate at which power is reached did not improve to the same extent. The lack of a significant time × group interaction effect on peak power and average power parameters may reflect progressive isometric loading, even though the two training programs have different mechanical and stabilization requirements. However, in TRX-based isometric training, instability and the need for multi-joint body control increase stabilization demands and may limit high strength and power production by the agonist muscles. In contrast, although dynamometer-based isometric training produces high, controlled torque, contractions occur at fixed joint angles and zero movement velocity, so this may not directly affect the speed component of power production. From this perspective, the two approaches may not have shown clear superiority over one another in anaerobic power development.

On the other hand, the significant main effect over time in the estimated 1 RM maximal strength value suggests that both training approaches may effectively enhance maximal strength production capacity. A review of the literature indicates that isometric strength training positively affects dynamic strength performance and can increase maximal strength not only under isometric test conditions but also through its positive impact on overall performance (Oranchuk et al., 2019). The two different isometric strength training methods created a need for higher strength production over time. This finding is consistent with studies on the fundamental training mechanisms that explain maximal strength development in the lower extremities. Suspension training improved 1RM values; similarly, while eight weeks of stable and unstable resistance training showed significant time-dependent effects on maximal strength, the study indicated that balance performance alone did not drive these effects (Soligon et al., 2020; Kibele and Behm, 2009). The lack of statistically significant improvements in the time × group interaction may stem from differences in load-velocity profiles across regions due to structural differences in training approaches, which may not fully reflect adaptations. This is because, in our current study, we assessed the estimated 1RM value using a load-velocity-based method rather than a direct 1RM test. The literature indicates that load-velocity-based 1RM estimates are used to observe general changes in strength; however, compared with directly measured 1RM tests, they are reported to contain estimation errors stemming from exercise, load, velocity, and methodology (Greig et al., 2023). Based on our findings, the hypotheses we established at the beginning of the study were not supported. The results showed that the different mechanical characteristics of the two training approaches were not sufficient to produce the predicted performance improvements through chronic adaptation; therefore, rather than assuming a general superiority of one training method over the other, training processes should be evaluated based on the performance variable and the loading method used.

One of the strongest aspects of our study is the direct comparison, within a randomized design, of two isometric training models differing in implementation and mechanical characteristics on isokinetic, balance, and anaerobic power performance variables in trained athletes. Conducting our study with active athletes enhances its applicability to training for athletic performance and at the competitive level. At the same time, objective laboratory measurements of isokinetic strength, muscle asymmetry, balance, anaerobic power, and estimated 1RM maximal strength enabled a multidimensional examination of the two training approaches. In particular, one methodological strength of our study is determining participants’ individual isometric contraction angles relative to the peak torque during isometric training in the dynamometer-based training group, as well as the minimum threshold required to perform the movement. Joint angle, muscle length, contraction intensity, and contraction duration influence performance-related adaptations in isometric training (Oranchuk et al., 2019). Building on these strengths, in future studies involving functional suspension systems such as TRX and isometric exercises performed on dynamometer devices, it is recommended to increase sample size, expand the number of comparable groups (such as control groups), quantitatively classify and compare training scope and intensity, and examine comparisons of training approaches that incorporate neurophysiological components such as cognitive load and neurocognitive mechanisms. Additionally, because of its portability and ease of use, TRX suspension training equipment may offer a more practical and feasible option in field conditions than laboratory-based training equipment, which has limited accessibility.

## 5 Conclusion

As a result, we observed significant main effects of time in isokinetic peak torque, OSI and MLI, anaerobic peak power, average power, and estimated 1RM maximal strength parameters following eight weeks of TRX- and dynamometer-based isometric strength training in athletes. In contrast, we observed no significant main effect of time for muscle asymmetry, API, or time to peak power. Furthermore, we found no statistically significant group effects or Time × Group interactions across all performance parameters.

Essentially, the authors explained that adaptations from isometric training have a temporally significant effect on performance-specific responses such as strength, balance, asymmetry, and anaerobic power measured in our study. Therefore, it appears more appropriate to select the method for TRX and dynamometer-based isometric training not solely based on equipment type, but also on performance characteristics, training content, and application conditions. These findings offer insight into isometric loading for both TRX-based functional exercises and dynamometer systems; however, they show no statistically significant difference between the two programs in time-dependent changes in the performance variables examined.

### Limitations

Our study has some limitations. First, the 8-week training period and the sample size of 16 young male athletes limit the certainty of the findings when evaluating differences between the two training programs. Based on the sensitivity analysis with the current sample size, the time × group interactions are sensitive to detecting relatively large effect sizes. Therefore, non-significant time × group interactions should not be interpreted as indicating that there are no smaller differences between training programs. At the same time, the fact that the participants were young male athletes limits the generalizability of the performance parameters to female athletes, different age groups such as adolescents and children, and individuals at different performance levels. Additionally, although the study evaluated many performance outcomes, it did not define a single primary or secondary outcome variable beforehand. For this reason, interpret the results of multiple statistical comparisons with caution. Furthermore, the study’s failure to use physiological measurements such as electromyography (EMG), muscle architecture, or tendon stiffness that could directly shed light on neuromuscular mechanisms limits the interpretation of the adaptation processes underlying the observed performance changes. Finally, using the estimated 1RM method based on the load-velocity relationship for maximum strength assessments is a methodological limitation compared with directly measured maximum strength readings.

## Data Availability

Data supporting the findings of this study may be requested from the corresponding author upon submission of a reasonable justification. The data are not publicly available due to the inclusion of sensitive personal information.
