# Supplementary material for: Comparative effects of suspension-based and dynamometer-based isometric training on neuromuscular performance in trained male athletes: a randomized comparative study
Source: Front Physiol. 2026 Oct 6;17:1959307. doi: 10.3389/fphys.2026.1959307 (PMC13680181; doi:10.3389/fphys.2026.1959307)
Supplement: Supplementary file 1 [file SupplementaryFile1.docx]

**Figure Supp Material.** TRX Position Holding Movements
